# Time-Frequency Analysis of Barkhausen Noise for the Needs of Anisotropy Evaluation of Grain-Oriented Steels

**DOI:** 10.3390/s20030768

**Published:** 2020-01-30

**Authors:** Michal Maciusowicz, Grzegorz Psuj

**Affiliations:** Department of Electrical and Computer Engineering, Faculty of Electrical Engineering, West Pomeranian University of Technology, 70-310 Szczecin, Poland; michal.maciusowicz@zut.edu.pl

**Keywords:** nondestructive testing, magnetic Barkhaunsen noise, magnetic anisotropy, time-frequency representation, signal processing, data mining methods, grain oriented steel

## Abstract

The paper presents a new approach to obtain information on magnetic anisotropy in Si–Fe grain oriented ferromagnetic steel based on the observation of the magnetic Barkhausen noise (MBN). Until now, in the literature one can only notice the MBN study of magnetic anisotropy in steels carried out in a single time or frequency domain. However, due to the observed high variability of the dynamics of the MBN phenomenon over its occurrence period, depending on the steel properties, the idea of utilization of combined time and frequency representations to obtain new or supplementary information arises. For this purpose, the MBN phenomenon was observed in various directions for steels with oriented magnetic properties. Then, using the short-time Fourier transform, time-frequency (*TF*) distributions were determined and features vectors enabling the quantification of crucial information were determined. Before performing the final experiments, a series of tests were carried out for different measuring conditions. As a result, it was possible to adjust the conditions enabling us to obtain the highest possible sensitivity for MBN and discrimination level between directional properties in the material. Then, an algorithm of detailed analysis and division of the *TF* representation into subranges was proposed, enabling the extraction of more detailed information about the phenomena occurring during the magnetization process. This allowed us to clearly indicate and then separate three areas of MBN main activity. Finally, the obtained angular distributions of selected features were presented and discussed, and further conclusions were given.

## 1. Introduction

Materials with magnetic properties depending on the direction of the magnetic field are called anisotropic materials [1]. Such structures have a preferred directions, called the easy magnetization axis, for which relatively lower magnetic field results in reaching magnetic saturation. Magnetic anisotropy can occur in a material for several different reasons, being intrinsic, extrinsic or induced [1]. The presence of anisotropy in steels is mainly related to their magnetocrystalline structure, grain shape and stress state [1,2,3]. Anisotropic properties can be introduced to materials by various methods. Among them one can distinguish: magnetic annealing, magnetic irradiation, plastic deformation, stress annealing, and electric field [1,4,5]. In industrial applications, the field of forming a specific properties of the material at its surface is covered by the surface engineering techniques. One of the main methods is the cold rolling process. Nevertheless, regardless of its source, magnetic anisotropy strongly influences the shape of the hysteresis loop as well as the coercivity and remanence. In consequence, it plays a significant role in the design of magnetic material for the needs of the electrotechnical industry. The steel with anisotropic magnetic properties is manly used in the machinery industry for construction e.g. transformers or electric motors [6,7,8,9,10]. Considering the relationship between magnetic and mechanical properties of steel, the evaluation of magnetic anisotropy is also used in the process of mechanical state assessment [8]. 

There are various methods of measuring the anisotropic magnetic properties such as: the rotational, torque measurement, magnetic hysteresis loop measurement and induced magnetic field measurement [1,11,12]. Recently, the possibility of determining directional magnetic properties by magnetic Barkhausen noise (MBN) has also been studied [13,14,15,16]. The MBN is related to reorganization of the magnetic domain structure of ferromagnetic material occurring under alternating magnetic fields [17,18,19]. The transformation process of the domain structure results in discontinuous changes in the magnetization of the material. The discontinuity originates from defects in the crystallographic structure of the material, e.g. dislocations, second-phase particles or phase and grain boundaries. Those pinning sites have a direct impact on the course and dynamics of Barkhausen effect. Considering all those aspects, MBN becomes a natural solution for the evaluation the magnetic materials anisotropy.

As mentioned earlier, the anisotropy of a given material is influenced by a number of factors depending on its microstructure, shape, machining process or plastic deformation. Ultimately, the characteristics of global magnetic anisotropy are determined by the resultant value of all factors affecting heterogeneous magnetic properties in different directions of a given material [20]. The impact of individual factors on the scale of magnetic anisotropy has been the subject of many studies, in which the dynamics of the magnetization process using the angular dependence of the MBN activity were observed [13,20,21,22]. Most often, this study concerned the evaluation of anisotropy associated with the magnetomechanical effect resulting from the effects of stress or plastic deformations occurring in materials (stress anisotropy) [23,24]. An important aspect, especially in the context of industrial applications, are the works devoted to the evaluation of magnetic anisotropy associated directly with the crystallographic structure of the tested material (magnetocrystalline anisotropy, MCA) [16,20,24]. Equally important are the results of works on the evaluation of anisotropy, induced during the machining process related to the rolling of materials referred to as rolling magnetic anisotropy (RMA) [13,15,20].

In earlier works, during the study of angular dependence of the MBN response, whole signal bursts measured within a half of magnetization period were analysed. These studies showed the potential for applying the Barkhausen effect to assess magnetic anisotropy [13,16,20,22,25,26,27]. In [24], the authors studied influence of the crystal and stress (residual and applied) anisotropy on the characteristics of maximum amplitude of the MBN envelope. The strong competition between both examined factors according to the stress state was presented as the main reason of the specific behaviour of the maximum MBN amplitude curve. In consequence, the interaction was assumed to have direct impact on the magnetic easy axis. It was shown, that at the low stress values the easy axis was related to crystal anisotropy, thus this causes the domains to rotate in the direction of the magnetic easy axis. However, at higher applied stresses, domains are forced to rotate into direction of new easy axis controlled by the direction of the applied stress. These findings were also referred to in [28], where the authors studied angular dependence of root-mean-square (RMS) of MBN for samples subjected to various thermomechanical processing stages (hot rolling, hot band annealing, cold rolling and final annealing). They documented comparable (to the aforementioned) relationships for partially recrystallized electrical steel. In [23], similar observations were carried out for samples with various grain size and strain state. The authors compared the results of monitoring of MBN activity (by number of MBN peaks counts, RMS and peak voltage value) obtained by three laboratories confirming a direct relationships between the material state and MBN. The impact of steel crystallographic texture was also investigated in [27], where a clear change in magnetic anisotropy was presented depending on the degree of cold-rolling. In [14], the authors examined the bulk magnetic anisotropy in pipeline steel, presenting three major reasons responsible for the magnetic easy axis: texture, microstructure and plastic deformation. They found difficulties in expressing correlation between the crystallographic texture and the angular dependence of MBN activity, denoting the plastic deformation introduced during the cold rolling process as the forming factor of magnetic anisotropy. The possibility of magnetocrystalline anisotropy evaluation was examined in [20]. It was shown that MBN could be a potential technique for the assessment if the effect of the residual stress would be eliminated. However, the results obtained mostly concern the influence of singe aspect at a time. Thus, they did not allow for a detailed analysis of the factors simultaneously affecting the resultant anisotropy, as the final angular distribution of the obtained Barkhausen noise activity depend on the dominant component. Therefore, recently the idea of magnetization dynamics study based on the definition of bands of MBN expressed in terms of time (time-bands) or magnetic field (*H*-bands) has been introduced and presented in several works [29,30,31]. The authors undertook a detailed analysis of the dynamics of phenomena occurring in samples made of pipeline steel. It was shown that by division of MBN burst into bands and processing the separate analysis of angular dependence of MBN energy distributions for each subperiod, it is possible to reveal precise information about the magnetizing processes being affected by various anisotropy defining factors. According to the presented results and discussion, at least the three different processes of the magnetization dynamics could be distinguished while their activity depends on the magnetization angle. The first one refers to the reversed domain nucleation bonded with the magnetocrystalline anisotropy energy, while the second relates to the main MBN peak and concerns the 180° domain walls (DWs), and the third one the 90° DWs motion. It was stated that structural features referring to microstructural banding along with grain shape and orientation identified with the RMA were probably affecting most the process of the second defined band. The RMA was discussed as that having an influence also on the third subperiod [30]. In [17], the authors additionally examined MBN resulting from both hysteresis and initial magnetization process. They noticed that the angular distribution of the amplitude of the MBN envelope obtained for the hysteresis process differs from that achieved for initial magnetization. This is explained by the influence of the domain nucleation process on the motion of 180° domain walls. 

Even though the works provide promising results, the main problem of the proposed methodology is the precise definition of the time or magnetic field bands (sections) as it is not clear [31]. The authors underline the influence of various conditions, not only related to the anisotropy factors, on the achieved MBN characteristics. Nevertheless, in all the papers presented above, the MBN analysis was carried out in the time domain, what may limit the information concerning the processes dynamics and courses. Additionally, only a few parameters regarding the energy (or RMS), peak amplitude or number of peaks where considered during analysis. However, due to the complexity of the MBN signals observed for anisotropic steels, it is reasonable to analyse the signal more deeply. Therefore, in this paper MBN properties expressed in both the time and frequency domains, which allows us also to observe the dynamics of the signal changes, were considered. Time-frequency transformation methods have found several applications for Barkhausen signal analysis in various applications until now [32,33,34,35,36,37]. However, to the authors’ best knowledge, the TF methods have not been used yet to analyse the Barkhausen effect for the needs of magnetic anisotropy assessment. Recently, a possibility of extended time-frequency (*TF*) processing using short-time Fourier transform (STFT) for the need of MBN signal analysis was presented in detail [32]. That paper gives the overview of *TF* transformation application to the MBN study. The published work provides also a detailed discussion on the utilization of the *TF* analysis and compares the content of information brought by *TF* features with the classical analysis (obtained in single time or frequency domains). Thus, this paper shows the approach to evaluate magnetic anisotropy in electrical steels based on the analysis of the MBN signal considering only its *TF* representation. The *TF* domain allows analysis of the change of the signal dynamics (referring to the range of signal’s frequency band) in a successive time or magnetic field related periods. This may lead to gain additional information [32,33,34]. The following sections of this paper present the experimental setup and measuring methodology, along with the electrical grain-oriented steel samples used and the procedure of *TF* transformation of the MBN signal. A further section refers to the successive steps of the *TF*-based MBN activity analysis method and anisotropy evaluation. In the final section of this paper, the conclusions are drawn.

## 2. Procedure of the Experiment

This section presents details on the preparation of the measuring system, the test object, and the measuring procedure itself. Additionally, the procedure used to transform the data into the time-frequency domain and to calculate the features of the received *TF* representations will also be presented.

### 2.1. Experimental Setup

**Figure 1:**
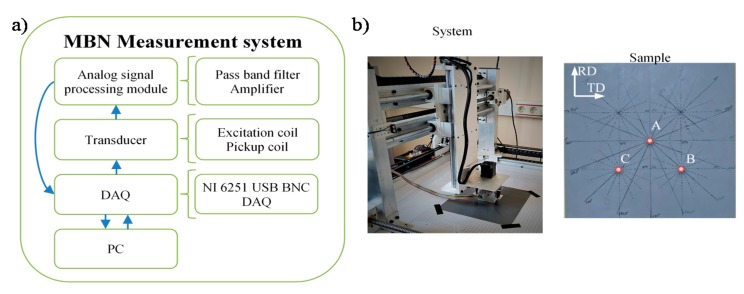
Magnetic Barkhausen noise (MBN) measurement system: (**a**) block diagram, (**b**) photo of the setup and sample.

The operating scheme of the measurement system is presented in Figure 1. All experiments were carried out using a transducer having two subsections: the magnetizing and the pick-up ones. The magnetizing coil was wound on a C-shaped ferrite core while the pick-up coil placed between the pole pieces of the yoke gathered the Barkhausen noise signal (*U*_BN_). The measurements were controlled by a personal computer through the data acquisition board (DAQ). Before driving the coil, the sinusoidal magnetizing waveform was generated using a digital-to-analog (D/A) converter and then amplified. Next, the *U*_BN_ signals were bandpass filtered, amplified, and acquired using analog-to-digital (A/D) converter. The band of the filters has been set for the range of (0.6–97.5) kHz. The excitation frequency *f*_E_ of 10 Hz, utilized during the experiment, was determined based on a preliminary test conducted for a series of frequencies (details given in Section 3.1). The sampling frequency *f*_S_ was set to 250 kHz and resolution to 16 bits. The XYZ scanner was used to shift and rotate the transducer over the examined sample. Springs were used to maintain constant mechanical pressure on the measuring head. As a result, the lift-off during the measurements was not greater than 0.2 mm (corresponding to the thickness of the tape securing the transducer plane). The details of the system can be found in [32,38].

### 2.2. Measurement Procedure

**Figure 2:**
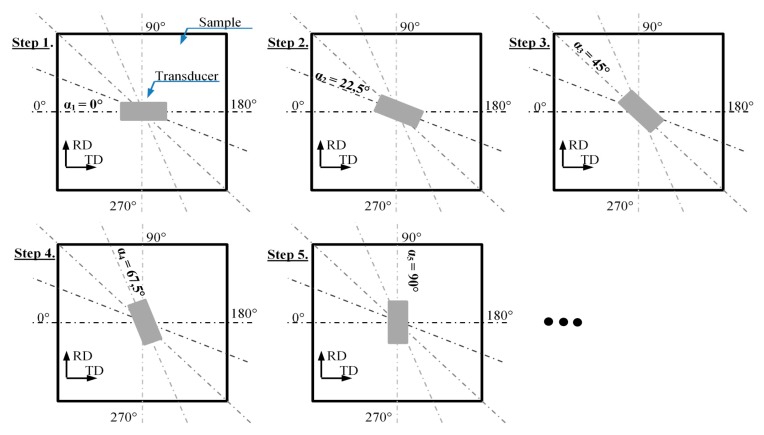
View of the first 5 successive steps of the transducer alignment at a given position on the sample.

**Figure 3:**
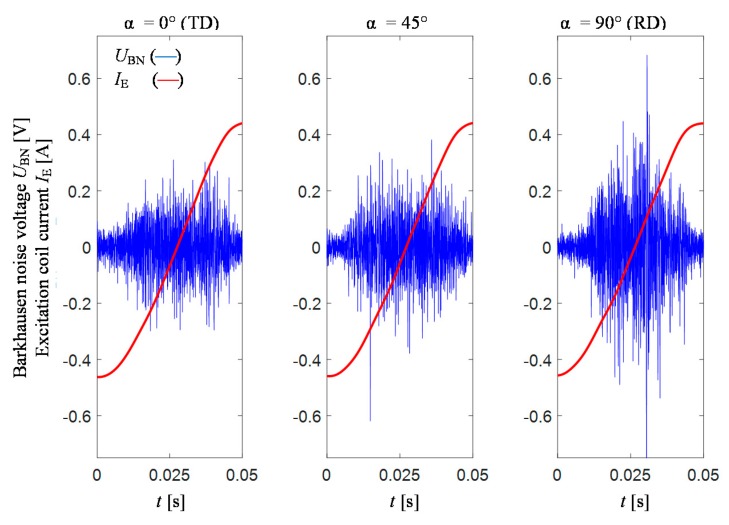
Exemplary distributions of MBN voltage U_BN_ and magnetizing current I_E_ acquired for excitation frequency of 10 Hz and voltage amplitude of 0.275 V.

The test were carried out on conventional grain-oriented Si–Fe electrical steel of a thickness of 0.27 mm with negligible roughness of surface (enabling full contact with the transducer). The photo of the sample with depicted rolling direction (RD) and transverse direction (TD) along with the view of the measuring procedure is presented in Figure 1. The experiments were carried out in two stages: preliminary and final. During the preliminary stage, the measuring conditions were adjusted in order to obtain high sensitivity for material changes and further high discrimination rate between the analysed parameters of *TF* representation of the MBN computed for different locations of the transducer over the tested sample. Then, the final examination was performed using the selected conditions. All measurements were made at three different locations on the sample depicted in Figure 1b as: A, B and C. In order to evaluate the anisotropy distribution, at each mentioned location on the sample, a 360 degrees rotation of the MBN transducer (over its symmetry axis) was undertaken. As a result, 16 measurements of MBN signals were made between 0 and 360 degrees in angular steps α_step_ of 22.5 degrees. For each transducer’s angular position, 10 successive MBN measurements were taken, each allowing collection of signals of 10 magnetizing periods. The initial 5 successive steps of measuring procedure were visualized in Figure 2. First, the measurements were taken along TD (0 degree angle between the TD and the MBN transducer). The exemplary results of *U*_BN_ along with the magnetizing current *I*_E_ obtained for the 0, 45 and 90 degrees position of the transducer with respect to the TD and for the magnetizing frequency of 10 Hz are presented in Figure 3. One can clearly notice the difference in the course and dynamics of the observed Barkhausen effect depending on the alignment of the transducer. The highest activity, especially in term of the main MBN peak, can be observed in the case of 90° direction (RD).

### 2.3. Computational Procedure of Time-Frequency Data

First, the transformation of all acquired MBN data into the *TF* domain was carried out. For that purpose, the STFT transformation was utilized, as it allows uniform computational division of the *TF* space to be achieved [39]. The STFT-based *TF* representation is mostly used for the spectral analysis and processing of non-stationary signals under various applications for e.g., audio signals [40], medical electromyography (EMG) and electroencephalography (EEG) signals [41] or for speech recognition [42]. The STFT representation is composed of the Fourier transform results obtained for subsequent time periods of the signal [32]. Each time, a given signal interval and a window function are taken into account. The time spans of successive computational iterations frequently overlap widely. Thus, the STFT result can be characterized by the high redundancy of information contained in the data. By application of the STFT transformation, the complex *TF* representations *S*_BN_(*t*, *f*) of *U*_BN_ and furthermore their spectrograms |*S*_BN_(*t*, *f*)|^2^ were achieved. The parameters of the computational procedure were obtained based on tests carried for a selected range of window size, following a similar procedure to that described in [32]. The key criterion in the selection of window width was to obtain the highest resolution over time still enabling separation of MBN activity areas according to the analysis procedure described in next section. Finally, calculations were made using the Kaiser-type window function (with the parameter of the Bessel function equal to 0.5) of 128 samples, which corresponds to a period of 512 µs and frequency band Δ*F* of 1952 Hz. In order to increase time resolution, the window overlapping was used with the step of 0.75. In consequence, the effective time resolution Δ*T* was equal to 128 µs. 

Considering multiple acquisition of the MBN signals during several magnetizing periods, the smoothing procedure was applied, which resulted in average spectrogram distribution for a given orientation denoted as *BN*_TF_S_. Next, in order to quantify information provided by the spectrograms, a multiple-feature extraction was applied and vectors of several features were obtained for each individual measurement case. Several of the *TF* parameters refer to some statistical properties. In that case, the information was extracted using various forms of mean values (i.e., arithmetic, geometric, etc.) centroid, variance or standard deviation, skewness or kurtosis. Additionally, the shape of the *TF* spectrogram, and its energy distribution or entropy was also analyzed. Finally, the obtained vector of *TF* parameters allows the description of various characteristics of the *TF* representations such as symmetry, shift of the centre in *t*- or *f*-axis, flatness, uniformity or monotonicity, etc. The definition and properties of all proposed *TF* features as well as the utilized computation procedure was introduced earlier and discussed in detail in [32].

## 3. Time-Frequency Representation Results, Data Analysis and Discussion

An important part preceding the analysis of the properties of data contained in the *TF* domain of the MBN signal and the assessment of the range of anisotropy is the determination of measurement conditions. The dynamics of the Barkhausen phenomenon largely depends on the frequency and amplitude of the magnetizing field. Consequently, their values affect the energy distribution in the *TF* representation and the ability to observe its changes. Therefore, before making a proper evaluation of anisotropy, it is necessary to determine the conditions that create the possibility of obtaining adequate sensitivity to the assessed aspects of steel. During the determination of the measuring conditions, it is also necessary to take into account the possibility of a clear indication of the MBN activity areas. In the following sections, the results of the selection of testing parameters, the procedure for the extraction of MBN activity areas and, finally, the results and analysis of the anisotropy of the tested steel are presented. 

### 3.1. Determination of Measuring Conditions of Magnetic Barkhausen Noise (MBN)

Before final measurements, various measuring conditions were applied and the quality of the information provided by spectrograms was analysed in the context of a high discrimination level between data obtained for three angular orientations of the examined sample. For that, several levels of the magnetizing field amplitude and frequencies were used in two preliminary stages. In the case of the first, 10 different values of excitation voltage ranging from 0.2 V up to 0.7 V were considered. This allowed to provide various levels of magnetizing conditions ranging from around 1.4 kA/m up to 4.5 kA/m. Measurements were made for two frequencies: 1 and 10 Hz. As a result, it was possible to monitor the range and character of changes in the *TF* distributions at low and relatively fast dynamics level of the MBN effect. In the second stage, the measurements were conducted at a single level of magnetizing field for 7 frequencies: 0.5, 1, 2, 5, 10, 20 and 30 Hz. In both stages for each case the measurements were carried out at a single location on the sample for transducer aligned at 0, 45 and 90 degrees to the TD. 

**Figure 4:**
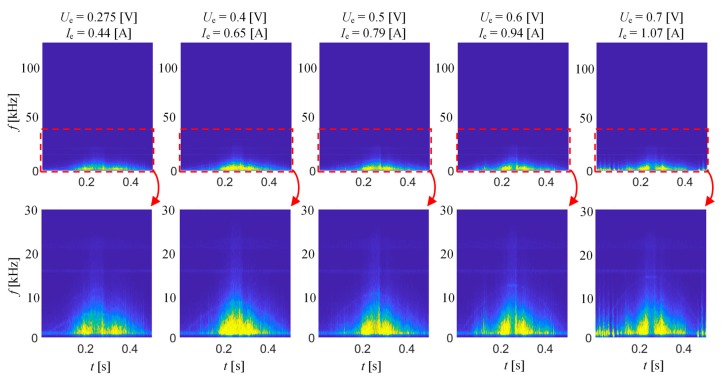
BN_TF_S_ spectrograms obtained for excitation frequency of 1 Hz and various excitation voltage level.

**Figure 5:**
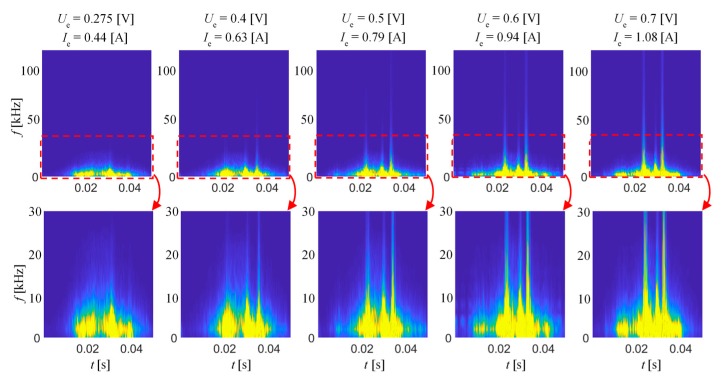
BN_TF_S_ spectrograms obtained for excitation frequency of 10 Hz and various excitation voltage level.

Figure 4 presents selected spectrograms obtained for different level of magnetizing field at a frequency of 1 Hz, while Figure 5 presents 10 Hz for the RD direction. Both results sets were divided into two parts. The first one (top row) shows the full frequency band of the *TF* spectrograms, while the second provides the close-up (up to 30 kHz) of the selected range. For comparison of the results, all were plotted using the common color range. As can be seen, when current value (and so the field strength) is increasing, the arrangement of MBN activity areas is changing. The MBN intensity increases with the current value, affecting the visible increase in the frequency band in the corresponding time bands of subsequent *TF* representations. For low field values, the MBN signal can be described basically as a continuous spectrum characteristic over MBN time. However, above a certain field level, one can notice the formation of clear time sections in which the frequency band significantly increases. These bands are separated by time periods in which MBN activity is clearly lower. As the current increases, the larger MBN frequency band can be observed in the main activity areas. This phenomenon is more visible for excitation frequency of 10 Hz as the dynamics of the magnetization is faster, and thus the discrimination of MBN activity bands is easier to process.

**Figure 6:**
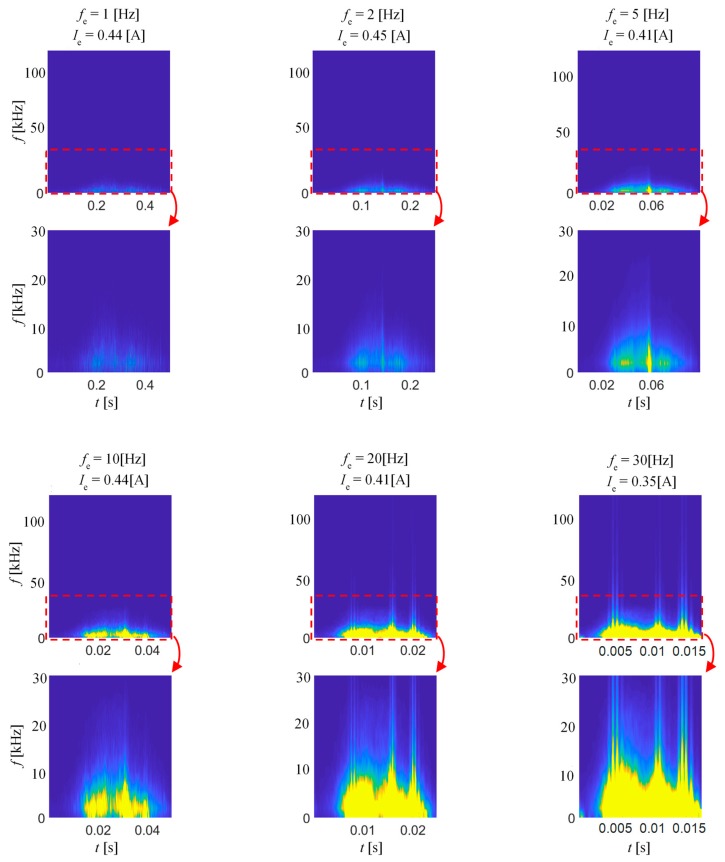
BN_TF_S_ spectrograms obtained for various excitation frequency and constant excitation voltage amplitude; view of the full frequency range (top) along with the close-up up 30 kHz.

Figure 6 shows the results of the second stage of the preliminary experiment. The spectrograms were obtained for six frequencies of magnetizing field between 1 Hz and 30 Hz. The sinusoidal signal voltage source was set to a constant value of 0.275 V. In consequence, as the frequency increases, the excitation current decreases. Nevertheless, although the magnetic field strength is decreasing along with frequency, the dynamics of the magnetization process is obviously increasing, resulting in a significant increase of MBN spectral activity both over time span and frequency range. The increase of the excitation frequency results in the increase of the rate of magnetic structure reorientation, which increases the spectral power density values that are observed on the spectrogram. However, at the same time, a clear decrease in the discrimination of the earlier described time bands representing the highest MBN activity can be seen. Additionally, in the high-frequency regime, the dynamics of the magnetization process is affected greatly by the eddy current effect. Therefore, considering all those aspects and relatively low thickness of the sample (0.27 mm), in order to obtain high level of distinguishing its various magnetic stages, a frequency of 10 Hz and a voltage of 0.275 V (allowing to obtain field of 1.8 kA/m) were selected for further experiments. 

### 3.2. Extraction of Barkhausen Noise (BN) Activity Regions

**Figure 7:**
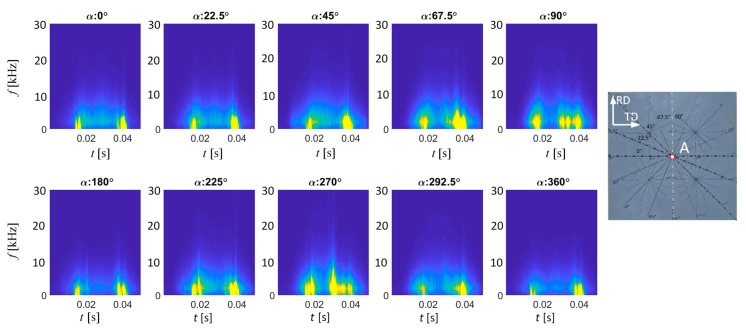
BN_TF_S_ spectrograms distributions obtained for various orientation of the transducer over location A in the sample.

As per the previous section, the final experiments were carried out for three different locations on the sample during full rotation of the transducer in steps of 22.5°. Figure 7 presents the spectrograms obtained for location A for selected 16 angular orientations of the transducer within the 0–360° range. Similarly, as in the case of preliminary results, all were plotted using the common color range. The change in spectrograms’ distribution with the change of angle is clearly visible, which allows one to easy indicate the activity bands. The largest variations in results can be observed between axis lying along 0–180° (TD and the hard magnetization axis) and along 90–270° (RD and easy magnetization axis). Starting from the angle of 0° of the transducer orientation, one can notice clearly the outlined areas of local high MBN activity in spectrograms. The first of these areas appears around 15 ms (which is the time before zero-crossing stage of the magnetizing current and corresponds to coercivity; see Figure 3). As the transducer rotates towards the RD and the easy magnetization axis (90 degree angle), this area is expanding both in time span and frequency as well. 

**Figure 8:**
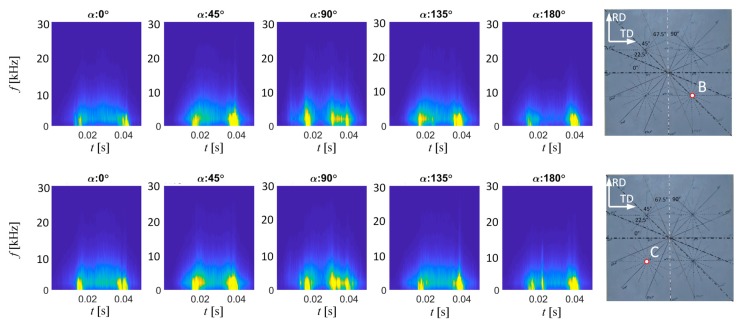
BN_TF_S_ spectrograms distributions obtained for various orientation of the transducer over B and C locations in the sample.

Another area of high MBN activity can be found close to the time of 40 ms (which refers to the period following the highest MBN peak stage) presenting similar behavior to the first one described. It is also visible on each spectrogram obtained for angles between TD and RD. However, the most significant difference can be noticed in reference to the *TF* distribution obtained for the RD direction. In the middle part of the time period covered by the spectrogram (around 30 ms), another, third high activity area is appearing. This corresponds to the time band of the main peak of the MBN (Figure 3). According to expectations, when analyzing the results for appropriate pairs of the transducer’s angular alignment for given sample’s direction, one can notice the repetitive characteristics in the *TF* distributions. The lower activity level of MBN is obtained for α equal to 0° and 180°. On the other hand, a significant increase in the activity is visible in case of α equal to 90° and 270°. The greater activity level for 90° and 270° is achieved due to the fact that there is a greater number of 180° type DW in the RD in the sample [15,16]. Similar observations were made for the two other locations (B and C) on the examined sample (Figure 8). Nevertheless, one can notice the two areas of the highest activity regardless of the transducer orientation, as well as the presence of an additional, third area (between the two previous ones) in the case of orientation along or close to the RD axis. This follows the observations presented by other researchers. Considering the analysis carried out in the time domain reported in the literature, it should be noted that the results obtained allow us to clearly identify the activity bands. To the best knowledge of the authors, in all disseminated works adequate indication of the time spans was described as the most problematic approach. Under these circumstances, the proposed methodology seems to be promising for carrying out the anisotropy analysis.

**Figure 9:**
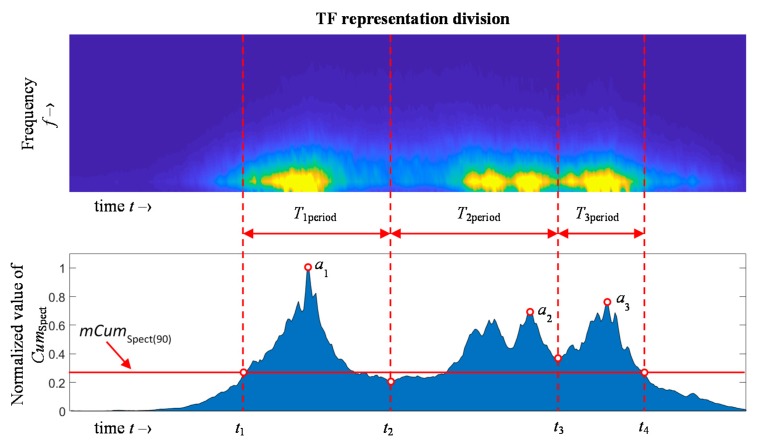
Visualization of the BN_TF_S_ spectrogram distribution and the 1D instantaneous cumulative spectrogram Cum_Spect_ with depicted time bands of MBN activity obtained for α equal to 90° (along RD axis).

In order to conduct the analysis, allowing quantitative evaluation of the information provided by the spectrograms, further data processing was proposed. Thus, for the purpose of the precise indication of the time bands limits, the activity of the MBN for successive time moments were accumulated along the frequency axis forming the 1D instantaneous cumulative power spectral *Cum*_Spect_ plots:

(1) $$Cum_{Spect}\left(t\right)=\sum_{i=1}^{f_{H}}BN_{TF_{S}}\left(t,i\right),\;t=0\ldots 50ms$$

where *t* refers to the time vector and *f*_H_ refers to the upper frequency range limit of the spectrograms equal to 100 kHz. The plot visualizing the calculation procedure is presented in Figure 9. The *Cum*_Spect_ distribution allows us to enhance, more clearly observe and indicate the areas of greatest MBN activity.

According to the initial qualitative analysis carried out for spectrograms achieved for all angular orientations of the transducer, the three areas of activity could be distinguished, which is in agreement with the results reported in the literature. In order to localize the time bands *t*_1_, *t*_2_, *t*_3_ and *t*_4_ of the successive MBN activity subperiods common to *TF* spectrograms for each angular case, the *TF* spectrogram obtained for the RD was considered, as it allows us to provide the clearest distinction between the three areas. As a result, the 1D distribution *Cum*_Spect(90)_ for α equal to 90° was calculated. Next, its median value *mCum*_Spect(90)_ was calculated. Furthermore, by comparing the *mCum*_Spect(90)_ with the value of the successive time moments of the *Cum*_Spect(90)_, the starting point (*t*_1_) of the first activity region (*T*_1period_) and the final one (*t*_4_) of the third region (*T*_3period_) were calculated. Then, the two remaining time bands, *t*_2_ and *t*_3_, were determined. First, the initial locations of inner bands were determined to be in the time position after the peak value of the first activity area and before the peak value of the last activity area, allowing the time range to be split into 3 subperiods. Then, three global maximum value *a*_1_, *a*_2_, and *a*_3_, one in each successive subperiod, were found. Finally, the times of occurrence of two global minima (*t*_2_ and *t*_3_) inside the time ranges defined by the location of *a*_1_, *a*_2_, and *a*_3_ were calculated. In consequence, the values of the *t*_1_, *t*_2_, *t*_3_ and *t*_4_ were used as common to indicate MBN activity ranges on *TF* spectrograms obtained for all angular alignment of the transducer.

### 3.3. Evaluation of Anisotropy

According to the analysis of works described in the introduction section, the distinguished three bands can be referred to various phenomena. The first refers to the magnetocrystalline anisotropy energy, while the RMA was reported as affecting the most the course of the second band and was discussed as that influencing the third subperiod too. Thus, the separate analysis of each subperiod can bring different information about the inspected material. The procedure carried out allows us to analyse the MBN response using time-frequency representation. The presence of the first period (*T*_1period_) before the major MBN peak can be linked to the time interval in which the domain nucleation appears during the reversal magnetization process. The second period (*T*_2period_) refers to the time interval of the main maximum peak of the MBN signal, which is characterized mainly by 180° DW movement. According to the earlier works, the time period assigned to *T*_3period_ can be associated with the movement of 90° DW. Therefore, based on the (*t*_1_, *t*_2_), (*t*_2_, *t*_3_) and (*t*_3_, *t*_4_) division, for each subperiod the set of *TF* features were calculated from each spectrogram.

**Figure 10:**
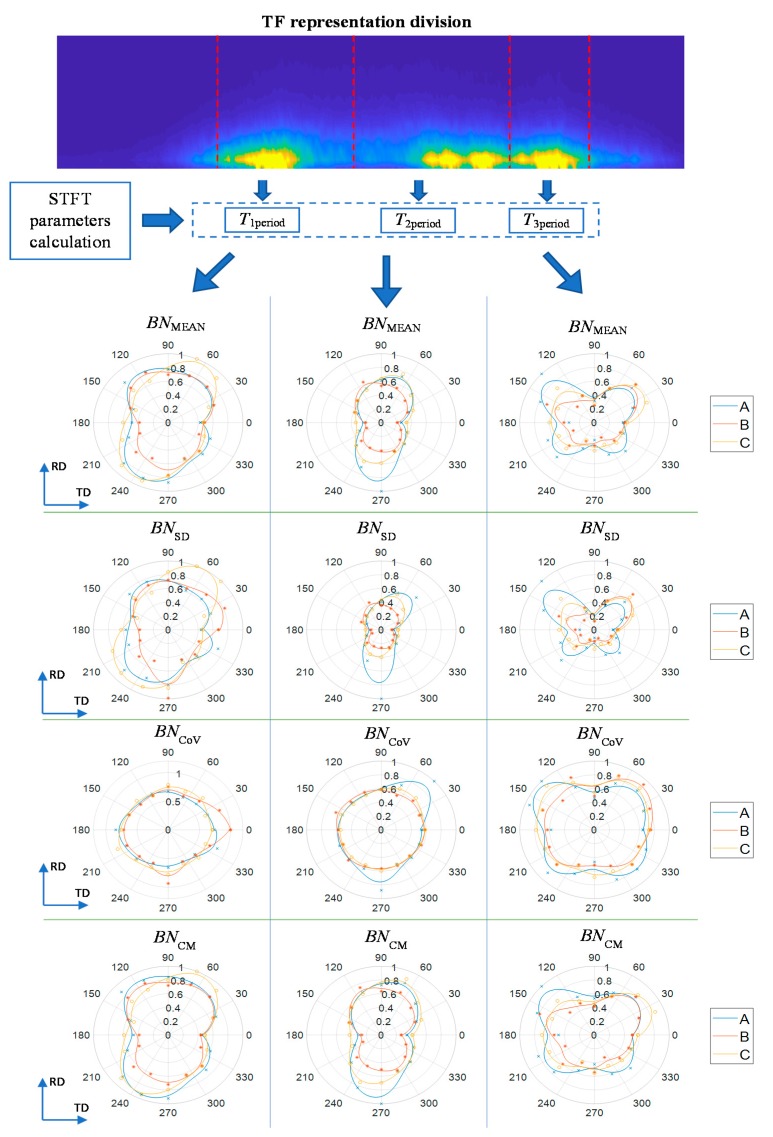
Selected time-frequency (*TF*) features angular distributions in three MBN activity subbands obtained for three locations (A, B and C) at the examined sample.

Angular distributions of selected *TF* features obtained for all three bands are presented in Figure 10: the mean *BN*_MEAN_, the standard deviation *BN*_SD_, the coefficient of variation *BN*_CoV_ and the concentration measure *BN*_CM_ coefficient. One can notice a good correlation between the distributions of the first two features mentioned. The *BN*_CoV_, which express the ratio of the first two features, confirms the good agreement of the those characteristics. The *BN*_CoV_ presents inconsiderable variance within the full rotation for the first two periods (*T*_1period_ and *T*_2period_). Based on the analysis of the variability of the *BN*_MEAN_ and *BN*_SD_ value for *T*_2period_, a significant difference between the values in TD and RD axes can be seen. Starting from the TD direction, the both features’ values generally increases with angle until the angular range close to the vicinity of the RD is reached and then begin to decrease forming the angular distribution close to an eight-like shape. The observed nature of the course results directly from an increase in the level and variability of values in this time interval. The growth of the MBN activity (larger *TF* space covered by high energy level) within this whole period for RD finds its confirmation also in the *TF* distribution concentration measure *BN*_CM_. The feature refers to distance measure procedures and is not sensitive to small quantities in distribution. It reaches higher values for more uniform distribution of the energy over the spectrum [32]. In the result of higher activity of MBN obtained for RD, generally the energy level is growing, simultaneously being more spread out over whole *TF* space of the *T*_2period_. This has an effect on lower concentration rate (greater distribution of energy) of the spectral distribution. Thus, the *BN*_CM_ value is getting higher. Summarizing, one can clearly indicate the rolling direction RD. Following a rough analysis, one can notice that generally the parameters distributions for the first time interval *T*_1period_ assume similar forms as the ones for the *T*_2period_ band discussed above. However, despite the high convergence of the characteristics, one can notice differences in the rate and dynamics of changes obtained in the case of *BN*_MEAN,_ *BN*_SD_ and *BN*_CM_ features. These characteristics obtained for the first period of activity show a relatively smaller difference in the value between TD and RD compared to those obtained for the second area. In addition, it can be seen that these changes occur with relatively slower dynamics as well, which can be seen in particular by comparing the distributions of features in the range between 30 and 150°. Similar observations can be made for a range shifted by 180° (around 270°). The observed courses of the angular characteristics are probably the effect associated with megnetocrystalline distribution under the low magnetizing field conditions. Under such conditions, the Barkhausen phenomenon is mainly influenced by 180° domain walls, and as the value of the field used increases, the contribution of nucleation of reverse domains and the 90° domain wall motion increases [43]. However, this observation should still be confirmed with additional experiments. The parameters for *T*_3period_ take higher values for intermediate angles between RD and TD forming butterfly-shaped distributions. One can also notice that the results present some discrepancies in the characteristics obtained for individual locations on the measurement sample. This may be due to a slight angular shift between the sample edges and the actual rolling direction of the sample. As a result, due to the relatively large distance between individual locations of measuring points, a slight angular shift between the characteristics may occur. Undoubtedly, the finally level of the magnetizing field strength used also has an impact on the error rate. On the one side, at a relatively low value, high distinguishability of individual states of the magnetization process were obtained, which was crucial for observation of activity subbands. At the same time, lower *U*_BN_ response voltage values were also registered, which had an impact on greater sensitivity to interfering factors and, as a result, led to greater measurement errors. Another factor that could affect the resulting divergence is the degree of repeatability of directional properties in different areas of the sample. Nevertheless, despite noticeable discrepancies and errors, the results seem to be promising in terms of the further evaluation of the anisotropy problem. Following the proposal for the analytical procedure that has been presented, it should be underlined that this allows straightforward definitions of time-bands angular distributions depicting various characteristic of material properties assessment. As a result, this can be utilized as a simple methodology for the material properties analysis and indication of the easy and hard magnetisation axis as well. 

## 4. Conclusions

This paper presented a new approach enabling the assessment of steel anisotropy based on time-frequency analysis of MBN signal. The proposed procedure provides a simple way of separating subperiods of the measured response of Barkhausen noise. The MBN *TF* analysis method presented above is an alternative way of obtaining information about directional properties in steels to the classical time-domain approach. The proposed methodology allows one to indicate precisely the bands of the MBN activity intervals. That is crucial in the subbands-based procedure. Additionally, bands definition was reported to be the major problem during the analysis. Therefore, the proposed method can be an interesting alternative. Considering the results obtained, it is believed that the approach can create the basis for the analysis of many factors affecting the resultant anisotropy of a material. As discussed earlier, various factors affect the MBN activity during the successive stages of a single magnetizing period. Therefore, the procedure can be utilized in the evaluation of the possibility of those factors’ influence on the resultant anisotropy. As a consequence of the proposed analytical procedure and obtaining an indication of individual areas of MBN activity, the opportunity arises to develop methods of a broader analysis of magnetic anisotropy in materials. The presented analysis in *TF* domain can be helpful in describing the changes in the magnetic domain structure behavior under various factors affecting anisotropy distribution in the ferromagnetic materials. Based on analysis of the spectrograms, specific frequency bands can be observed in given the subbands. However, it must be still deeply analyzed in further experiments, considering the influence of many other factors. Nevertheless, the method allows the determination of RD, TD axes and intermediate angles as well. 

In the future, it is assumed that research will be carried out using the samples simultaneously subjected to various factors, that influence the outcome of the material anisotropy and perform a wider verification procedures proposed by the techniques of material structure analysis. Then, the *TF*-based analysis procedure proposed in this paper is going to be correlated with the classical analysis methods in time domain. Additionally, as the relatively large variability of dynamics under various measuring conditions was clearly observed in this work, the study of the possibility of simultaneous utilization of multiple magnetizing parameters will be carried out. Then, it is planned to use the information gathered under several conditions to optimize the effectiveness of the properties evaluation.
